# A Method for Custom-Contoured Cushion Fabrication Based on Pressure Mapping for Wheelchair Users to Prevent Pressure Ulcers: Feasibility Quasi-Experimental Study

**DOI:** 10.2196/68612

**Published:** 2025-05-23

**Authors:** Alma De León-Hernández, Adriana Martínez-Hernández, Isabel Bolivar-Tellería, Andrea Bosch-Sánchez, María Fernanda Cabrera-Padilla, Carlos Omar López-López

**Affiliations:** 1 Institute of Applied Research and Technology (InIAT), Universidad Iberoamericana Ciudad de México, Prolongación Paseo de la Reforma, 880, Lomas de Santa Fe, Mexico City, 01219, Mexico, 52 5559504000 ext 4690; 2 Biomedical Engineering Program, Department of Engineering Studies for Innovation, Universidad Iberoamericana Ciudad de México, Mexico City, Mexico

**Keywords:** pressure ulcers, pressure map, custom-contoured cushion, wheelchair, wheelchair user

## Abstract

**Background:**

Pressure ulcers constitute a major health care burden, characterized by significant morbidity, diminished quality of life, and elevated treatment costs. Wheelchair users are predisposed to pressure ulcers due to sustained ischial and sacral interface pressures resulting from prolonged periods of sitting. Implementation of pressure-relieving interventions, including specialized seating systems engineered to redistribute load and augment the weight-bearing surface area, is critical for mitigating the risk of pressure ulcers.

**Objective:**

This study aimed to evaluate a methodology for the custom fabrication of pressure relief cushions, through the user-cushion interface pressure mapping to reduce high-pressure areas and increase contact area in wheelchair users.

**Methods:**

First, a validation study was carried out with 7 healthy volunteers. The pressure was determined with an FSA sensor (BodiTrak BT1510, Vista Medical Manufactures), and the cushion profile was obtained through a linear relation with pressure values. In the second phase, 10 cushions for wheelchair users were manufactured and tested. The resulting data from buttock pressure using a flat foam, Jay X2 (gel-foam), ROHO high profile (air), and customed-contoured cushions were analyzed and compared using the following 4 variables: peak pressure, peak pressure index, mean pressure, and contact area.

**Results:**

In the validation study, the statistically significant difference between the flat and the custom-contoured cushion showed a better performance in pressure relief for the custom cushion (mean pressure 27.3, SD 4.5 mm Hg and 34.6, SD 3.5 mm Hg; *P*<.001). Regarding the study with wheelchair users, custom-contoured cushions had lower peak pressure (mean 91.3, SD 36 mm Hg), peak pressure index (mean 69.5, SD 33.7 mm Hg), and mean pressure (34.2, SD 17.4 mm Hg) against flat, Jay X2, and ROHO high profile cushions (*P*<.005). The contact area (mean 1457.6, SD 254.1 cm^2^) was greater for the contoured cushion (*P*<.001) than for flat and ROHO high profile (Permobile) cushions; nevertheless, it was not significantly different from Jay X2 (*P*=.59).

**Conclusions:**

The main finding is that the buttock pressure mapping method produces custom-contoured cushions that, compared with commercial cushions, have good pressure distribution and increased contact area. These results suggest that pressure mapping is a good alternative for manufacturing affordable custom-contoured cushions that can prevent the development of pressure ulcers.

## Introduction

Wheelchairs are a critical assistive technology, enabling mobility and participation for individuals with physical disabilities worldwide. The World Health Organization (WHO) has estimated that 90 million people require a wheelchair to assist their mobility. Yet, only 18 million people can access an appropriate wheelchair [1]. Official statistics for Mexico in 2024 indicate that 8.3 million individuals live with a disability, with 3.3 million experiencing difficulty walking or rising from a seated position using their legs [2]. Furthermore, 4.1 million of this population lives in poverty [3]. The established correlation between poverty and disability in developing countries underscores the necessity for accessible, low-cost assistive technologies that are easily maintained and replaced.

Wheelchair users, who remain seated for prolonged periods, are considered a high-risk population for developing pressure injuries [4]. Pressure injuries, also known as pressure ulcers (term used in this work), decubitus ulcers, or bedsores, are localized lesions affecting the skin and soft tissues, resulting from sustained pressure and mechanical stress, typically over bony prominences or due to medical devices or personal items such as mobile phones [5]. These ulcers occur in 70% of cases at the sacrum, ischial tuberosity, and greater trochanter (pressure sites associated with the seated position). However, they may also develop on the occiput, scapula, elbow, heel, lateral malleolus, shoulder, and ear [6]. The etiology of pressure ulcers is multifactorial, involving a complex interplay of sensory deficits, alterations in skin moisture balance, and shear forces. The prevalence of pressure ulcers varies depending on underlying pathology, age, and comorbidities among wheelchair users. Notably, a systematic review and meta-analysis found that approximately one in 3 individuals with spinal cord injury develops pressure ulcers, highlighting the significant burden of this condition in this population [7].

One of the most widely used therapeutic strategies to mitigate the development of pressure ulcers is the use of pressure-relieving cushions for wheelchairs. These cushions are specifically designed to reduce focal pressure over bony prominences while increasing the contact area [8]. Various materials have been tested for fabrication, including foam, gel, air, and combinations [9-11]. In addition, dynamic cushions, which modify their shape through actuators such as pumps, have been developed to further alleviate pressure in high-risk areas [12,13].

While air and gel cushions have demonstrated efficacy in load distribution, they can be susceptible to damage, compromising their performance [14,15]. Consequently, the costs associated with repair and replacement can be prohibitive, limiting accessibility for many wheelchair users [9]. Custom foam cushions present a viable alternative for mitigating high-pressure areas [16]. However, inconsistencies in manufacturing processes and the complexity of mold generation can impede their widespread production. Furthermore, conventional fabrication techniques may pose risks to wheelchair users due to potential exposure to high-temperature chemicals that come into direct contact with the individual during the molding process. In addition, traditional molding is often time-consuming, and retaining information about the seat shape can be challenging due to the storage requirements for the molds [17].

The implementation of digital methodologies for shape capture and model generation in cushion fabrication facilitates the automation, acquisition, and storage of critical data, including cushion contour, pressure distribution profiles, and dimensional characteristics, streamlining and optimizing the manufacturing process. The pressure mapping of the user-cushion interface is a digital method for capturing the body shape, in which pressures are transformed into depth. The contoured cushion is produced through computer numerical control (CNC) foam carving. The main differences reported in the literature are the filters and smoothing of the pressure signals, as well as the relationship between the cutting depth and the interface pressure values, generally based on the deformation of the foam as a function of pressure [18]. Results have shown better pressure distribution performance in custom cushions than in flat cushions [19,20]. However, a comprehensive study has not been reported covering the design of the custom-contoured cushion through interface pressure, CNC manufacturing, and performance evaluation compared with the flat cushion and other cushions of different materials with the same pressure relief goal.

The objective of this study was to evaluate a methodology for the custom fabrication of pressure relief cushions, through the user-cushion interface pressure mapping to reduce high-pressure areas and increase contact area in wheelchair users.

## Methods

### Methodology

This work aimed to develop and validate a methodology for fabricating relief pressure custom cushions based on pressure maps of the user-cushion interface. Hence, the methodology is divided as follows: (1) pressure data collection, (2) surface model, (3) cushion fabrication, (4) validation study, and (5) cushion performance analysis and user satisfaction.

### Pressure Data Collection

The pressure maps of the user-cushion interface were obtained using the commercial mat BodiTrak BT1510 with a 16x16 matrix of pressure sensors. The calibration kit of the mat (ACC16-Standard Small Calibration Jig) was used to calibrate regularly to ensure the accuracy of the measures. Before measuring a subject, initial tests with a constant known weight were performed; if the pressure data had atypical values, the calibration process was conducted.

The participants were asked to sit on the testing cushion: a commercial high-density polyurethane foam block (R-90) of 35 mm, covered with the pressure-mapping mat. Seat depth and footrest height were adjusted according to the anthropometrical dimensions of the subject to ensure a neutral seating position (hips, knees, and ankles at 90°). Pressure at the buttocks was recorded every minute for 10 minutes. If a located point reached the maximum value (200 mm Hg) during the test, the data set was discarded, and the mat and subject were repositioned, to conduct a new test.

The procedure explained above was used to generate the contoured profile of the manufactured cushions. However, the pressure maps obtained with the flat foam block and 2 commercial pressure relief cushions were also used to evaluate the performance of the manufactured cushion. For every participant, pressure maps were obtained with a flat R-90 foam block, a ROHO High Profile cushion (air), and a Jay X2 cushion (foam-gel). Finally, the same procedure was conducted with the custom-contoured cushion fabricated using the proposed methodology to compare the performance.

### Surface Model

The pressure values obtained from the user-cushion interface with the testing cushion (pressure data collection section) were processed to generate the surface model of the contoured cushion. A linear relationship converts pressure values into cushion thickness values, establishing that the highest pressure, regardless of magnitude, corresponds to the lowest cushion thickness (40 mm). The areas where no pressure was recorded correspond to the points of the greatest foam thickness (75 mm). The surface was determined as follows:

 (A) Pressure measurements taken every minute for 10 minutes are averaged.


(1) $$P=(\sum_{i=1}^{10}p_{i})/10$$

where *i* is the number of the map taken every minute for ten minutes and $p_{i}$ are the pressure matrix


(2) $$P_{i}=\left(\begin{array}{ccc} P_{1,1} & \ldots & P_{1,16}\\ \vdots & \ddots & \vdots \\ P_{16,1} & \ldots & P_{16,16} \end{array}\right),$$

(B) The average pressure (P) is converted to a grayscale image ($P_{g}$).
(C) The contour of the seated person is defined through a threshold value to remove the pressure points out of the user-cushion contact area; then, a Gaussian filter is applied to obtain an offset ($O_{f}$) to soften the final profile of the cushion
(D) Two smoothing filters are applied to $P_{g}$ to increase the user-cushion contact area. First, a Gaussian filter, then the values are normalized. Finally, an averaging filter is applied to the normalized data ($P_{gN}$).
(E) The cushion profile is obtained according to the following relationship:

(3) $$Z=\left(\frac{Z_{M}-Z_{m}}{P_{gm}-P_{gM}}\right)P_{gN}+z_{M},$$

where 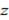 is the cushion thickness for each value in the 16×16 matrix in millimeters. $Z_{M}$ and $Z_{m}$ are the maximum and minimum cushion thicknesses, set to 75 mm and 40 mm, respectively. $P_{gM}$ and are the maximum and minimum smoothed and normalized grayscale pressure.

The offset $O_{f}$ is applied to the obtained surface *Z* according to:


(4) $$S=Z-O_{f},$$

where *S* is the final profile of the cushion.

### Cushion Fabrication

**Figure 1.**
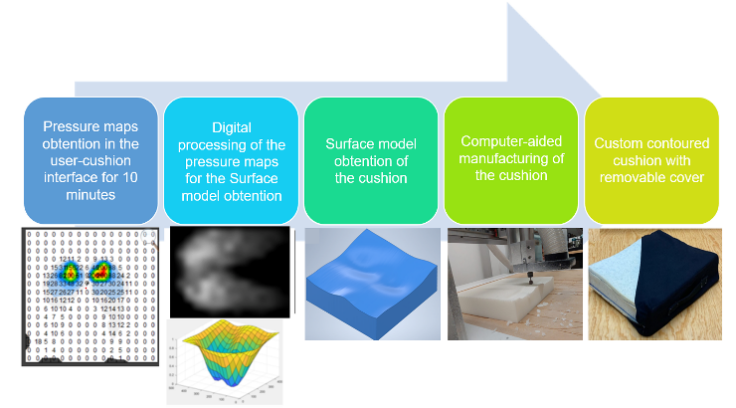
The schematic of the methodology developed to manufacture the custom-contoured cushion from the user-cushion interface pressure maps.

Once the surface model of the cushion was obtained (“Surface Model” section), the custom-contoured cushion was manufactured according to the following steps:

1. The values obtained with the procedure described in the section “Surface Model” were introduced into a computer-aided design (CAD) algorithm created in Inventor (Autodesk) to get the 3D model of the cushion.
2. The CAD model of the cushion was passed to a CNC milling machine (Shapeoko, Carbide 3D) to carve a commercial high-density polyurethane foam block (R-90) with dimensions of 45×45×7.5 cm to obtain the custom shape.
3. Once the foam has the desired shape, a sheet of 6.35 mm thick memory foam is added to each face of the foam.
4. A waterproof cover of a 1 mm layer of soft silicone (Smooth-On, Ecoflex 00‐30) is cured onto the memory foam.
5. Finally, a custom removable cover was manufactured using a cloth of 94% cotton and 6% elastomer. The full procedure is represented in the schematic of Figure 1.

### Validation Study

The present study consists of two clinical phases.

#### Performance Validation of the Manufactured Cushion Using the Pressure Mapping Methodology

Seven nonwheelchair-using volunteers were included, with the following inclusion criteria: age between 18 and 60 years, absence of pain that would prevent them from remaining seated during pressure mapping data acquisition (10 min), and full range of motion in pelvic limbs, allowing for proper positioning (hip, knee, and ankle at 90°).

#### Assessment of the Performance of Custom-Contoured Cushions with Wheelchair Users

In addition, 10 wheelchair users were included, meeting the following inclusion criteria: age between 18 and 60 years, absence of pressure ulcers within the past year, absence of pain that would prevent them from remaining seated during pressure mapping data acquisition (10min), absence of joint limitations or postural abnormalities that would impede proper positioning during pressure mapping data acquisition, and absence of communication impairments or intellectual disability that would hinder informed consent.

For both groups of participants, anthropometric measurements and wheelchair dimensions were collected to evaluate their suitability. Then, the procedure explained in the “Pressure data collection” section was conducted: First, the pressure maps of the user-cushion interface with a polyurethane foam (R-90) of 35 mm thickness were recorded for 10 minutes. Then, the same procedure was repeated with the Jay X2 (gel-foam) and ROHO high profile (air) cushions. Finally, once the custom cushion was fabricated with the flat foam maps, 10 pressure maps were recorded using the custom-contoured cushion for performance evaluation.

### Cushion Performance Analysis and User Satisfaction

The resulting data from buttock pressure using each cushion (flat foam, air, foam-gel, and contoured) was analyzed and compared using IBM SPSS software. The variables used to evaluate cushion performance were peak pressure (PP), peak pressure index (PPI), mean pressure (MP), and contact area. The pressure variables are reported in mm Hg and the contact area in cm^2^.

Peak pressure is the highest-pressure value obtained in each measurement. Peak pressure index is the mean of the peak pressure with the pressures surrounding it; the PPI indicates the pressure under the ischial tuberosity area [21]. Mean pressure is the average value of the pressures distinct from zero. Contact area is the total area of the cells with a pressure value different from zero, reported in cm^2^.

User satisfaction was evaluated using the Quebec User Evaluation of Satisfaction with Assistive Technology (QUEST 2.0), which includes 12 items to assess satisfaction with a specific assistive device.

- Eight items assess the characteristics of the assistive device, including dimensions (size), weight, adjustments, safety, durability, simplicity of use, comfort, and effectiveness.
- Four items assess service aspects, including service delivery, repairs and maintenance of the device, professionalism of service, and follow-up service.

Participants were asked to rate their satisfaction with the device and service on a 5-point scale ranging from 1 (“not satisfied at all”) to 5 (“very satisfied”) [22].

### Statistical Analysis

Descriptive statistics were performed for all variables, calculating means and SDs for quantitative variables, and frequencies and proportions for qualitative variables. Pearson correlation test was performed between anthropometric measurements and wheelchair dimensions of the users. Furthermore, medians were compared using Mann-Whitney *U* test to analyze pressure values and contact areas between the different cushions for all participants.

### Ethical Considerations

This project was approved by the Institutional Review Board at Iberoamerican University (project number. 174/2022), and all participants signed the informed consent. The data of the participating subjects are found only in their clinical records and are protected by the current personal data protection laws in Mexico, to which the Universidad Iberoamericana adheres. For this study, the data of the subjects were anonymized, and their only compensation was their custom-contoured cushions.

## Results

### Performance Validation of the Manufactured Cushion Using the Pressure Mapping Methodology

A total of 7 nonwheelchair-using volunteers were included in the validation of the manufacturing process and performance of the custom-contoured cushions. Pressure measurements were initially taken on a flat foam surface, then cushions were manufactured according to these measurements. Pressures (PP, PPI, and MP) and contact area were compared. Statistically significant differences were found in all measurements, with lower pressures and larger contact areas observed in the cushions manufactured using the developed methodology (Table 1).

**Table 1.** Comparison of pressure and contact area variables between flat foam and the manufactured custom-contoured cushion in nonwheelchair-using volunteers. Data are presented as mean (SD).

Variable	Custom-contoured cushion, mean (SD)	Flat cushion, mean (SD)	*P* value
Peak pressure (mm Hg)	75.5 (12.3)	101.0 (13.4)	<.001
Peak pressure index (mm Hg)	53.9 (12.4)	73.6 (14.8)	.005
Mean pressure (mm Hg)	27.3 (4.5)	34.6 (3.5)	<.001
Contact area (cm^2^)	1716.4 (128.8)	1509.5 (137.4)	<.001

### Assessment of the Performance of Custom-Contoured Cushions With Wheelchair Users

#### Wheelchair Users

A total number of 10 wheelchair users were included in the study, with a mean age of 47 (SD 12) years, of whom 6 (60%) were female. The most common diagnosis was spinal cord injury (3/10, 30%), followed by poliomyelitis (2/10, 20%), cerebral palsy (2/10, 20%), amputation (2/10, 20%) and cerebrovascular disease (1/10, 10%). The mean wheelchair usage time was 10 (SD 5) hours per day. None of the participants used pressure-relief cushions. Only 2 had a history of pressure ulcers and the most common comorbidity was hypertension (2/10, 20%) and diabetes (2/10, 20%).

To explore the correlation between a proper wheelchair prescription and the user’s wheelchair characteristics, the relationship between anthropometric measurements and chair dimensions was evaluated. This analysis revealed a statistically significant correlation only between pelvic width (mean 41.7, SD 6.5 cm) and chair seat width (mean 41.1, SD 4.7 cm*; r*=0.83; *P*=.005). Conversely, no correlation was found between seat height (mean 45.8, SD 7 cm) with leg length (mean 42.3, SD 4.4 cm*; r*=0.19; *P*=.67) or seat depth (mean 83.6, SD 9.6 cm) with buttock-popliteal fossa length (mean 43.1, SD 4.8 cm; *r*=0.42; *P*=.29).

#### Cushion Performance Analysis

The performance of the custom-contoured cushion was evaluated by comparing pressure distribution variables derived from pressure mapping data, including PP (mm Hg), PPI (mm Hg), MP (mm Hg), and contact area (cm²). These variables were compared between the custom-contoured cushion and 3 commercial cushions: flat foam, Jay X2 (gel-foam), and ROHO high profile (air). Statistically significant differences were found in all variables when comparing the custom-contoured cushion to each commercial cushion; the custom-contoured cushion exhibited lower pressure values and a greater contact area. The only exception was contact area when comparing the custom-contoured cushion to the Jay X2 gel-foam cushion, where no statistically significant difference was observed (mean 1457.6, SD 254.1 cm² vs mean 1442.4, SD 187.7 cm²; *P*=.595) (Table 2).

**Table 2.** Comparison of the custom-contoured cushion manufactured with the proposed methodology with a flat foam cushion and 2 commercial cushions, through the 4 evaluated metrics: peak pressure, peak pressure index, mean pressure, and contact area. Data are presented as mean (SD).

Variable	Manufactured cushion	Commercial cushion						
	Custom contoured	Flat foam		Jay X2		ROHO high profile		
	Mean (SD)	Mean (SD)	*P* value	Mean (SD)	*P* value	Mean (SD)		*P* value
Peak pressure, mm Hg	91.3 (36)	119.9 (37.5)	<.001	100.7 (42.6)	<.001	106.5 (51.8)		;<.001
Peak pressure index, mm Hg	69.5 (33.7)	96.3 (39.6)	<.001	79.5 (37.8)	<.005	83.9 (45.8)		<.001
Mean pressure, mmHg	34.2 (17.4)	41.2 (23)	<.001	40.7 (19.8)	<.001	41.2 (21.4)		<.001
Contact area, cm^2^	1457.6 (254.1)	1250.2 (264.8)	<.001	1442.4 (187.7)	.59	1357.1 (221.7)		<.001

### User Satisfaction With the Pressure Mapping–Based Custom-Contoured Cushion

User satisfaction was evaluated using the QUEST 2.0 questionnaire. The mean satisfaction rating for the assistive device was 4.2 (SD 1.07), while the service received a mean rating of 4.9 (SD 0.14). Among device aspects, adjustments and simplicity of use received the highest ratings (mean 4.7, SD 0.75). Furthermore, users reported a perfect score (very satisfied=5) for professionalism and follow-up service.

**Figure 2.**
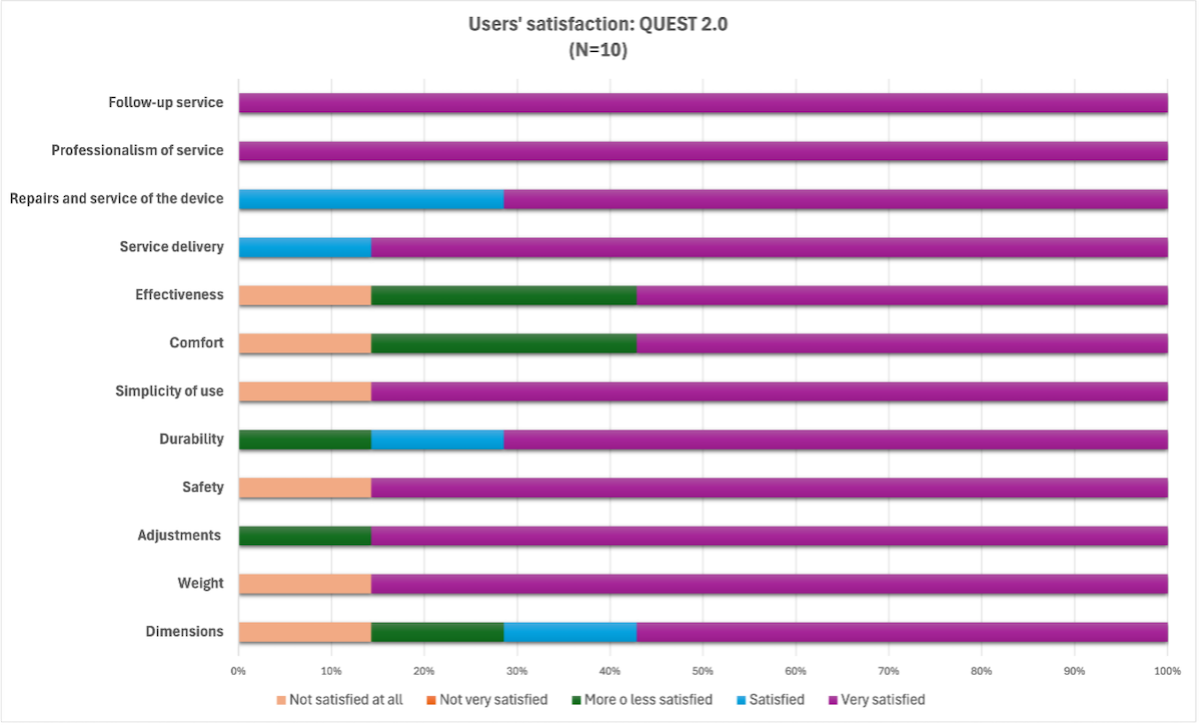
Users‘satisfaction according to Quebec User Evaluation of Satisfaction with assistive Technology (QUEST 2.0) questionnaire.

The distribution of user satisfaction related to the remaining eight items of the QUEST 2.0 questionnaire is presented in Figure 2.

## Discussion

### Principal Findings and Comparison With Previous Works

This work aims to develop and validate a methodology for fabricating relief pressure custom cushions based on pressure maps of the user-cushion interface. Two validation phases were conducted; first, 7 nonwheelchair-using volunteers were included to evaluate the performance of the contoured cushion versus a flat foam cushion. Then, 10 wheelchair users were included in the study to assess the performance of the custom-contoured cushion versus 3 commercial cushions by comparing pressure distribution variables, including peak pressure (mm Hg), peak pressure index (mm Hg), mean pressure (mm Hg), and contact area (cm²).

According to the results presented in Table 1, the performance of the manufactured custom-contoured cushion was better than that obtained with the flat foam, which was similar to that reported in the literature. Li et al [19] developed a custom-contoured cushion by converting the interface pressure under the buttocks into the carving depth, reporting a decrease in mean and PP under the buttocks against a flat cushion. Regarding the performance of the custom-contoured cushion against the Jay X2 and ROHO high profile, evaluated in the second phase of the validation study, the cushion manufactured with the proposed methodology presents lower PP, PPI, and MP. In the case of the variable of contact area, in our study, the best contact area is provided for the gel-foam cushion.

According to the literature, several studies have compared commercial cushions using the interface pressure. Sy and Tam [18] reported similar pressure relief characteristics between a custom-contoured cushion and ROHO and Jay 2 cushions; however, the recording time is not reported. As part of a rapid review, He and Shi [15] reported 3 articles using a Jay 2 and a ROHO high profile, reporting the pressure at ischial tuberosities between 94 to 160 mm Hg and 102 to 196 mm Hg, respectively, which is consistent with our results, the gel-foam cushion performs better than the air cushion. In the study by Gil-Agudo et al [11], a ROHO air cushion had lower pressure values than a gel-foam Jay cushion; the MP maps were obtained by averaging the data from 1.5 minutes of recording. Mendes et al [9] reported that in the static position, Jay Fusion Air showed the best rates for average pressure and contact area, while the Roho had the lowest average for the PP. The data were collected for 5 minutes, recording 10 frames per second.

However, user-cushion interface pressures captured using a pressure mat entails several challenges: the constant requirement to recalibrate the mat; its correct positioning under the user avoiding wrinkles or folds; high sensitivity to the user position and movement, which impacts the resulting contour; the user must wear smooth clothing, free of pockets, prominent seams, buttons, or any objects that generate high-pressure points; and finally, the time and frequency of pressure data capture, because they are important factors for the variables analyzed (MP, PP, PPI, and contact area). Regarding this last point, in the aforementioned studies, the time ranges from 1.5 minutes (without sampling frequency) to 5 minutes with 10 frames per second; in our case, the measurement duration was 10 minutes with one map per minute. The optimal duration of pressure measurements is still unclear and deserves further investigation.

On the other hand, this technique automates the user shape capture, avoiding manual, time-consuming methods and waste material. In the proposed methodology, the pressure values are converted into cushion thickness values by a linear relationship, in which it is established that the highest pressure, regardless of its magnitude, corresponds to the lowest thickness of the cushion (4 cm) and the lowest pressure corresponds to the highest thickness (7.5 cm), resulting in contours that have a thickness gradient that does not restrict the movement of the user.

Regarding to the relationship between anthropometric measurements and wheelchair dimensions, these parameters were intentionally examined, finding that only seat width and hip width had a statistically significant correlation. These results are unfortunate, as they reflect the limited access to custom-made wheelchairs in our country. These findings differ from the WHO’s definition of an “appropriate wheelchair,” which should meet the user’s needs, offer a good fit, be safe and durable, be locally available, and include maintenance services [23].

At Iberoamerican University in Mexico, we aim to provide a personalized cushion designed for good pressure distribution at an affordable cost, as an alternative to commercial cushions. The university owns the necessary infrastructure for manufacturing the cushions, including the pressure mat, data processing software, and CNC equipment, and the institution covers its associated costs. The materials needed to manufacture the cushion are as follows: a block of polyurethane foam (R-90), a sheet of memory foam, a soft silicone layer (Smooth-On, Ecoflex 00‐30), water-based contact adhesive (SILER), cotton cloth, and 2 zippers. The total cost for these materials amounts to US $50.

### Conclusions

In this study, custom-contoured cushions were manufactured by mapping buttock pressure according to a linear relationship, establishing that the highest pressure, regardless of magnitude, corresponds to the lowest cushion thickness. Seven cushions were made for 7 healthy volunteers, showing better performance in pressure relief against flat foam. Ten contoured cushions were manufactured for wheelchair users, and the peak pressure, peak pressure index , mean pressure, and contact area were determined. The custom cushions showed better pressure relief characteristics than flat foam R90, Jay X2, and ROHO high profile cushions. This methodology presents an affordable option for wheelchair users to obtain a pressure relief cushion.

## Abbreviations

CAD: computer-aided design
CNC: computer numerical control
MP: mean pressure
PP: peak pressure
PPI: peak pressure index
QUEST 2.0: Quebec User Evaluation of Satisfaction with Assistive Technology
WHO: World Health Organization
